# Cytotoxicity on low-grade canine meningioma with the use of somatostatin analog (octreotide): An in vitro study

**DOI:** 10.1093/noajnl/vdae111

**Published:** 2024-07-02

**Authors:** Maria Teresa Mandara, Alessia Tognoloni, Giuseppe Giglia, Massimo Baroni, Cristian Falzone, Pietro Calò, Elisabetta Chiaradia

**Affiliations:** Department of Veterinary Medicine, University of Perugia, Perugia (IT); Department of Veterinary Medicine, University of Perugia, Perugia (IT); Department of Veterinary Medicine, University of Perugia, Perugia (IT); Clinica Veterinaria Valdinievole, Monsummano Terme (IT); Clinica Veterinaria Pedrani - Diagnostica Piccoli Animali, Zugliano (IT); Polo Neurologico Veterinario, San Marino (RSM); Department of Veterinary Medicine, University of Perugia, Perugia (IT)

**Keywords:** chemotherapy, dog, meningioma, octreotide, somatostatin

## Abstract

**Background:**

Meningioma is the most common tumor of the central nervous system of dogs. For this tumor, surgery remains the treatment of choice, either alone or in combination with radiotherapy. Unfortunately, chemotherapeutic strategies are practically absent in dogs and palliative therapies are the only option to surgery. Somatostatin receptor subtype 2 (SSTR2) is expressed in canine meningioma. Since the potent cell-proliferation inhibiting effect of somatostatin (SST), the aim of this study was to investigate in vitro the effects of octreotide, as SST analog, in the viability of canine meningioma.

**Methods:**

Four surgical canine meningiomas were used in this study to establish cell cultures. Expression of SSTR2 was verified with immunolabelling in FFPE samples and cell cultures. The effects of octreotide on cell viability were assessed by 3-(4,5-dimethylthiazol-2-yl)-2,5-diphenyl-2H-tetrazolium bromide (MTT). After 24 hours they were exposed to different concentrations of octreotide (0.1 nM, 1 nM, 10 nM, 100 nM) for 24 and 48 hours.

**Results:**

All meningiomas consisted of grade I tumors. The cultured neoplastic cells expressed SSTR2 from 80% to 100%. Octreotide significantly increased cell death after 48 hours of continuous exposure, with 10 and 100 nM octreotide doses. The percentage of cell viability was 80.92 ± 4.9 and 80.49 ± 3.61, compared to the control, respectively, consistent with decreased cell viability of about 20% for both doses.

**Conclusions:**

Octreotide reduced the alive neoplastic cultured cells of low-grade canine meningioma in a dose-dependent pattern with continuous exposition for 48 hours. These results support an alternative systemic treatment of meningioma with octreotide in the dog.

## Lay Summary

Meningiomas are common tumors that grow from the protective covering around the brain and spinal cord. Currently, there are no effective drugs to treat meningiomas. In this study, researchers wanted to test whether a drug called octreotide could help treat meningiomas. They took meningiomas from 4 dogs and grew the tumor cells in the lab. Then, they treated the dog meningioma cells with different doses of octreotide for 24 and 48 hours. Their results showed that octreotide reduced the number of tumor cells by about 20% at the highest doses after 48 hours of treatment.

**Key Points**

Octreotide was able to decrease the cell viability of low-grade canine meningioma in vitro.
Good response of SSTR2 to octreotide was reported for low-grade canine meningioma.

**Importance of the Study**

This study opens new systemic therapeutic perspectives in the approach to canine meningioma. An alternative choice in the post-surgery tumor management of easier feasibility by owners compared to radiotherapy sounds of high social and economic impact. Moreover, alternative therapies are currently called for meningiomas refractory to radiotherapy and for those for which surgery is not applicable. Finally, it is important to look at this study according to a One Health approach to this tumor which increasingly corroborates as an animal model for human counterpart.

Meningioma is the most common tumor occurring in the central nervous system of the dog. Its incidence rates are on the rise probably due to the increased use of magnetic resonance imaging (MRI) and improved diagnostic protocol in pet medicine. According to the histological criteria derived from the WHO human classification, recently several veterinary neuropathologists reached a consensus on meningioma grades among dogs so that canine grades I, II, and III meningiomas account for 51.6%, 41.1%, and 7.3% of cases, respectively.^1,2^ In humans, WHO grade I tumors are significantly more prevalent, accounting for 80.1% of all meningiomas,^3^ followed by grade II and finally by grade III meningioma. For this tumor, surgery remains the treatment of choice^4,5^ either alone or in combination with radiotherapy. Systemic therapy, in the form of chemotherapeutic strategies, plays a minor role in the management of human meningiomas, practically nil in dogs.

Currently, palliative therapies with anticonvulsant drugs, corticosteroids, and/or analgesics are the only option for owners declining surgery or in case of surgically inaccessible meningiomas.^6^ However, for these cases prognosis remains poor.^6^ Unfortunately, data from chemotherapies and radiation therapies either alone or in combination with surgery are not consistent due to the general lack of histological diagnosis or detailed information included in the published studies.^6^ Moreover, the absence of established guidelines for drug dosing and the small case series included in the literature make the results significantly limited. What we do know is that the prognosis remains poor for dogs with primary brain tumors treated with chemotherapy alone. Similarly, for radiotherapy used as a monotherapy, it has been assessed that extra-axial tumors like meningioma have a more favorable prognosis compared to intra-axial neoplasia.^7^ On the contrary, for canine meningioma histologically diagnosed after surgery, radiotherapy yields a mean survival time of 16–30 months, compared to approximately 10 months for cases submitted to surgery alone.^6^ Additionally, the combination of hydroxyurea and imatinib-mesylate showed good efficacy.^8^ Interesting data have been reported in 11 dogs vaccinated with an autologous tumor cell lysate combined with synthetic toll-like receptor ligands after surgery. They consist of 645 days of median survival time compared to 222 days for surgically treated controls.^9^

More recently, the expression of somatostatin receptor subtype 2 (SSTR2) has been demonstrated in canine meningioma, in vivo as in vitro.^10^ This finding not only is the object of interest for diagnostic purposes in dogs, but it also opens new therapeutic perspectives using somatostatin (SST) analogs. In fact, it is known that somatostatin is a potent cell proliferation inhibitor. Binding to somatostatin receptors (SSTRs), somatostatin contributes to the regulation of tumor growth blocking the cell cycle and producing potential antiproliferative effects.^11^ Considering the potent effect and short half-time of somatostatin, synthetic SST analogs, like octreotide, pasireotide, and lanreotide have been used for experimental drug trials in human oncology.^12^

In this study we aimed to investigate the effects of octreotide (OCT) at different doses and treatment intervals in the viability of canine meningioma cell cultures, paving the way for potential future chemotherapeutic approaches to this tumor.

## Materials and Methods

### Study Design

Four canine meningiomas obtained during therapeutic surgery were used for this study. All of them were destinated to: (1) Histological diagnosis and grading, (2) Setting up of cell culture, (3) Culture cell typing and identification of SSTR2 expression, and (4) Viability tests on cell culture with octreotide.

### Inclusion Criteria

Inclusion criteria for the selected cases were as follows: (1) Magnetic Resonance Image findings referred to an intracranial or spinal cord extra-axial tumor consistent with meningioma, (2) Surgical excision identified as the first suggested therapeutic approach, (3) Size of tumor ≥1.5 cm for which MRI excluded significant necrotic or suppurative events, and (4) Histologically confirmed diagnosis of meningioma.

### Histological Diagnosis and Tumor Grading

After surgical removal, all 4 tumors were divided into 2 parts. One-half of any sample was fixed in 10% neutered buffered formalin and then paraffin-embedded for histological diagnosis and grading using H&E staining. 4 µm formalin-fixed paraffin-embedded (FFPE) sections of each tumor were also used for immunolabeling to type the tumor and to demonstrate SSTR2 expression. Immunohistochemistry (IHC) was performed with the standard streptavidin-biotin peroxidase complex method, according to the manufacturer’s protocol (Abcam, Cambridge, UK). To type the neoplastic cells, primary antibodies against Vimentin (mouse monoclonal; clone V9; 1:250; Dako, Milan, IT) and Epithelial Membrane Antigen (EMA; mouse anti-human EMA monoclonal; clone E29; 1:40; Dako, Agilent, Santa Clara, California) were used following published protocols.^13^ In addition, rabbit anti-rat SSTR2 polyclonal antibody (1:500; 4°C overnight; Alomone Laboratories, Jerusalem, Israel) was used as a primary antibody. Antigen retrieval was performed by microwaving at low power for 20 minutes, with citrate buffer at pH 6.0. Endogenous peroxidase was neutralized by a peroxidase block (3% H2O2 for 5 minutes). FFPE canine gastric fundus and pancreas samples were used as positive controls. 3-amino-9-ethylcarbazole and Carazzi’s hematoxylin were used as a chromogen and counterstain, respectively.

### Primary Culture of Canine Meningiomas

For each tumor, the remaining half was utilized to establish cell cultures according to previously published protocol.^9^ Briefly, after washing the tissue in Dulbecco’s phosphate-buffered saline (PBS; DPBS), without Ca^2+^ and Mg,^2+^ containing 100 U/mL of penicillin, 100 mg/mL of streptomycin and 250 mg/mL of amphotericin B (Sigma-Aldrich), blood clots and blood vessels were removed. Tumor samples were then dissected to obtain small pieces (1–3 mm) submitted to enzymatic digestion with 2 mg/mL trypsin for 10 minutes at 37°C and with 1.5 mg/mL of collagenase type IA (Sigma-Aldrich) at 37°C for addition 2 hours. Undigested tissue was removed using a 70 mm cell strainer (BD Biosciences, San Jose, California, USA). The cells were collected by centrifugation at 700 × *g* for 10 minutes, washed in DPBS, resuspended in Dulbecco’s modified Eagle’s medium supplemented with 10% fetal bovine serum, 100 U/mL of penicillin and 100 mg/mL of streptomycin (Sigma-Aldrich). After a alive cell count with Trypan Blue, cells were seeded at density of 20 × 10^3^/cm^2^ in T-flasks and expanded in monolayer culture at 37°C in a humidified atmosphere of 5% CO_2_. Non-adherent cells were discarded after 24 hours. Culture medium was changed every 48 hours.

### Culture Cell Typing and Identification of SSTR2 Expression

Cells were seeded at a density of 4 × 10^4^/cm^2^ on coverslips, placed in 6-well plates, and incubated at 37°C in a humidified atmosphere of 5% CO_2_ to about 60% confluence. Adherent cells were washed twice in PBS, fixed for 15 minutes at room temperature with 4% paraformaldehyde (pH 7.2), and washed again with PBS. To type culture cells and confirm SSTR2 expression, immunocytochemistry for vimentin, EMA, and SSTR2 was carried out, respectively, using the same protocol used for IHC.

### The Effects of Octreotide on Cell Viability

The effects of octreotide on cell viability were assessed by 3-(4,5-dimethylthiazol-2-yl)-2,5-diphenyl-2H-tetrazolium bromide (MTT). Briefly, cells were seeded at a density of 10.10^3^/well in 96 multi-well plates. After 24 hours they were exposed to different concentrations of octreotide (0.1, 1, 10, and 100 nM; Sigma-Aldrich) for 24 and 48 hours. After treatments, the medium was removed and MTT (0.5 mg/mL c.f.) was added. Dark blue formazan crystals, obtained after 2 hours of incubation at 37°C, were solubilized in DMSO, and the corresponding absorbance at 570 nm was measured using a microplate reader (Infinite 200 PRO, TECAN, Männedorf, CH). Cell viability results were expressed as the percentage of viable cells, obtained as a ratio between absorbance of treated wells/absorbance of control wells × 100. Data obtained from at least 4 different experiments performed in triplicate were recorded as mean ± SD (standard deviation). Untreated cells were used as a control.

### Statistical Analyses

Statistical significance was checked by performing an Analysis of Variance (ANOVA) with Bonferroni correction for multiple pairwise comparisons. *P*-values below .05 were considered statistically significant.

#### Ethic statements.—

This study was conducted in accordance with the VICH Topic GL9 (GPC) guidelines. The study utilized biopsy samples obtained during surgical excision proposed to the owners as the first treatment of choice, and then regularly submitted to histological diagnosis. Before surgery a consent/assent signed module is required by the clinical establishment to the owners informed about any risk related to surgery (eg, anesthesia, intra-surgery blooding or herniation, etc). No experimental surgical approach to the tumors will be part of the project. Therefore, the sample size issue does not fall into ethical implications.

## Results

### Histological Diagnosis, Cell Typing, and SSTR2 Expression

The 4 meningiomas were surgically removed from dogs aged between 4 and 9 years, 3 of which were female. Three of the tumors occurred in the frontal cortex, while the last one was in the proximal tract of the cervical spinal cord. Histologically, they consisted of meningothelial, microcystic, rhabdoid, and angiomatous subtypes. All tumors were histologically graded as grade I meningioma. At immunohistochemistry the neoplastic samples expressed both vimentin and EMA, in all the tumor area and from 70% to 90% of the entire tumor area, respectively. SSTR2 was expressed in all the tumors, from 60% to 70% of the entire tumor area.

**Figure 1.**
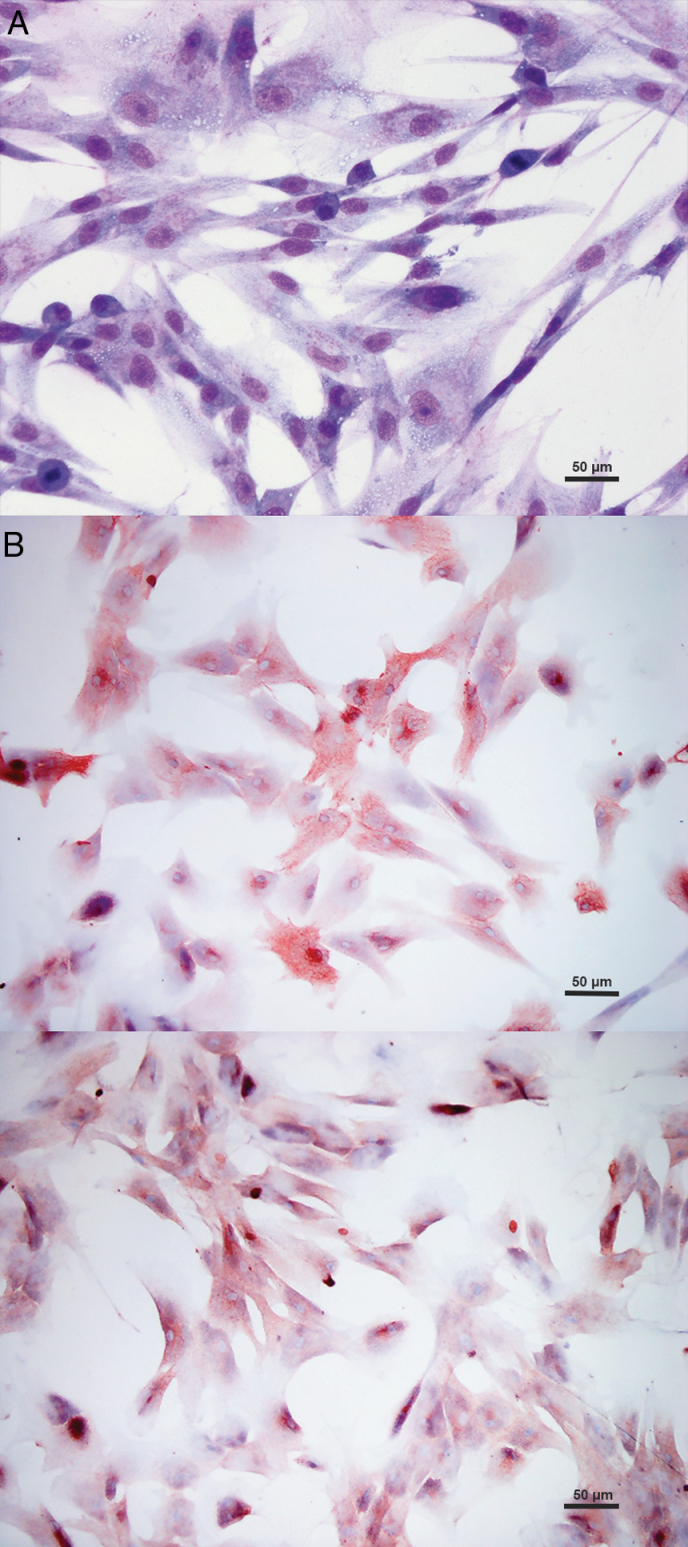
Primary cell cultures from canine meningioma. (A) Cultured cells tend to arrange in fusiform clumps. May-Grunwald-Giemsa stain. (B) Many neoplastic cells show cytoplasmic or membrane EMA immunoreaction (Immunocytochemistry). (C) Diffuse marked cytoplasmic SSTR2 immunoreaction of the cultured neoplastic meningeal cells (Immunocytochemistry).

Adherent spheres of fusiform branching-shaped cultured cells were obtained in primary cell cultures (Figure 1A). Immunocytochemistry performed on the primary cell cultures revealed immunoreaction for vimentin in 100% of cultured cells and immunoreaction for EMA (Figure 1B) in a cell percentage ranging from 40% to 95%. SSTR2 was immunoexpressed in all the primary cell cultures (Figure 1C). Cultured cells expressing SSTR2 ranged from 80% to 100%.

### Effects of Octreotide on Meningioma Cell Viability

**Figure 2.**
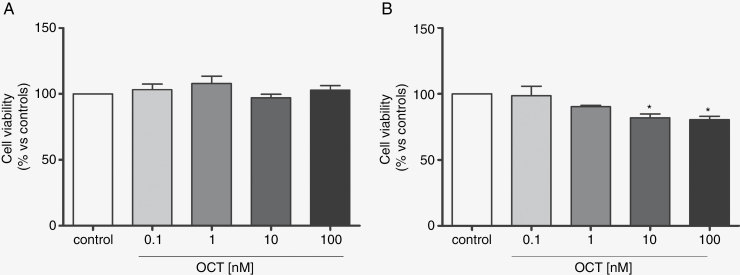
Cells were exposed to different concentrations of octreotide (OCT) for 24 hours(A) and 48 hours(B). Cell viability was measured by MTT assay. Data from 4 independent experiments performed in triplicate are expressed as the mean ± SD. Cell viability was calculated as the percentage of ratio between OD (optical density) of the samples treated with OCT and OD of control (*P < .05).

In order to define the putative toxicity of OCT on cultured cells obtained from canine meningioma, cell viability was evaluated after drug exposure for 24 and 48 hours at different doses. Octreotide produced no significant increase in cell death after 24 hours of treatment at all. Indeed, the cell viability observed at 24 hours of exposure to all tested doses of OCT was quite similar to the control. A significant increase in cell death was observed after 48 hours of treatment with the highest doses of OCT, namely 10 nM and 100 nM. In particular, compared to the control, the percentage of cell viability was 80.92 ± 4.9 and 80.49 ± 3.61, respectively, consistent with decreased cell viability of about 20% for both doses (*P* < .05; Figure 2). No significant effects were observed for 48 hours of treatment with 0.1 nM and 1 nM.

## Discussion

Somatostatin receptors (SSTRs) are differently expressed in human meningioma, with SSTR2 being the most commonly identified subtype.^14^ Its expression has been demonstrated also in canine meningioma.^10^ Activation of these receptors by somatostatin results in triggers intracellular pathways leading to cell cycle arrest.^15^ Besides a direct anti-neoplastic effect, the activation of SSTRs plays a crucial role in opposing secondary effects associated with tumor growth. In fact, SSTR activation promotes vasoconstriction, inhibits angiogenesis, modulates the immune response, and decreases secretion of growth factors, like vascular endothelial growth factor,^16^ with positive effects on the clinical presentation of the tumor. Compared to natural somatostatin, synthetic somatostatin analogs are characterized by less potent effect and longer half-time, and so their use is preferred. Octreotide and lanreotide are somatostatin analogs mainly binding to SSTR2.^17,18^

In this study, we investigated the effects of octreotide on the viability of canine meningioma cell cultures obtained from 4 grade I meningiomas. Cells were exposed to different drug concentrations for 24 and 48 hours. At 24 hours significant decrease in cell viability was not observed for any dose, while at 48 hours octreotide significantly reduced the alive cells with 10 and 100 nM doses, supporting the minimum of 20% as the cutoff value significantly decreased cell viability for both the doses.

In humans, studies in vitro of cytotoxicity have been performed using octreotide for meningiomas of different grades. A low number of cases (12%) was considered to be octreotide not-responsive and for the responsive tumors, cell viability was not significantly different in different grades. For 1 and 10 nM doses of octreotide, 26.55% and 27.5% viability were observed on the third day of treatment, respectively.^16^ Nevertheless, inhibition of cell proliferation was significant just at >10% in the majority of the tested tumors, without any correlation with WHO grade.^16^ Comparing these results with ours, it seems that canine meningeal neoplastic cells of low grade are more responsive than their human counterpart at 10 nM dose of octreotide, producing a significantly decreased cell viability (20%) just at 48 hours. At the same time, the cell viability tended to be constant with the much higher dose of 100 nM, simulating a certain type of drug resistance by SSTR2s at high doses. If a higher inhibitor effect on cell viability is reported in cells expressing more SSTRs^16^ and considering the high expression of SSTR2 up to 100% of the cultured cells of this study, it could be assumed that octreotide has a feebler antiproliferative effect in vitro canine meningioma than in human counterpart. Nevertheless, the significant correlation between octreotide response and SSTR2 expression has not been proved^16^ so that SSTR2 expression level sounds to be an inaccurate predictor of the octreotide response. In this regard, the functional status of SSTRs could possibly be considered to affect the effectiveness of the response.^19^

All the tumors used in this study were histologically consistent with grade I, including one rhabdoid subtype. Contrarily to humans in which rhabdoid meningioma is part of the group of grade III meningiomas due to the high rate of recurrence and death,^3^ in the dog, in the absence of biological data assessed in large cohorts of rhabdoid meningiomas, we are used to grade it strictly based on histological criteria.^1^

SSTR2 concentration is not significantly different from grades I to II in humans as in canine meningioma.^10^ For this reason, it can be not arbitrary to translate data obtained in grade I meningioma of the dog to grade II. In addition, it is useful to consider that in the absence of tissue adjacent to the tumor histological examination of benign biopsy samples could not identify invasion, which represents in itself a sufficient criterium for grade II tumor, enabling to underestimate grade II malignancy.^1^ Therefore, for all these reasons we can hypothesize that antiproliferative effect expected with octreotide treatment on higher malignancy tumor (grade II) of dogs could be not so far from those observed in this study. Moreover, it is important to highlight that in humans the WHO grade I tumor is the most frequent, accounting for 80.1% of all meningiomas,^3^ but despite benign histology, recurrence rates are not negligible and call for further efforts to improve the present grading schemes. On the contrary, due to the highest number of grade II meningiomas, in dogs recurrence is a common post-surgery event which cannot be ignored in the prognostic evaluation and in the tumor approach.^20^

As for grade I meningioma of dogs, meningothelial subtype tends to express SSTR2 more extensively compared with fibroblastic, transitional, and papillary subtypes^10^ almost similar to what is reported in humans.^16^ However, response to octreotide shows to be independent also from tumor subtype much more supporting to be independent of SSTR2 concentration.^16^

The use of somatostatin as a monotherapy for good neoplastic disease control is reported in human meningioma.^21^ This effect sounded most pronounced in WHO grade I tumors,^19^ while data obtained from studies on more aggressive meningiomas are still considered unsatisfactory.^22–24^ However, although in the last 2 decades numerous studies have been published on this topic, meta-analysis results have been regarded as compromised by poor quality of evidence and so as not definitely conclusive.^19^ More recently, combination between octreotide and everolimus, an mTOR inhibitor, has been investigated and its additive antiproliferative effect on human meningiomas of different WHO grades proved, especially in more aggressive tumors.^25–28^

Even though research on potential for treatment of human meningiomas with somatostatin analogs have improved and developed in the last years, the European Association of Neuro-Oncology has not provided guidelines regarding their use yet,^29^ and Norwegian guidelines consider somatostatin analogs for experimental treatment alone.^29^

To conclude, in this study, octreotide reduced the alive neoplastic cultured cells of canine meningioma in a dose-dependent pattern with continuous exposition for 48 hours. These results sound like a promising starting point for alternative systemic treatment of meningioma with subcutaneous^24^ or intramuscular injection^28^ of octreotide also in the dog, especially for low-grade tumors in poor clinical condition elderly patients, for surgery inaccessible masses, for recurrent tumors or tumors refractory to radiotherapy. Unfortunately, we have no data obtained with other chemotherapies in cultured cells from canine meningiomas of low grade useful for comparison. Anyway, endpoints need to be defined to translate these results into clinical trials and to measure response to somatostatin analog treatment in vivo. To this purpose, progression-free survival and partial radiological response on consecutive MRI investigations at a defined time apart in patients with no increased neurological signs or need for corticosteroids should be considered for future studies.^29^ Advanced scintigraphic imaging proving the expression of somatostatin receptors by meningioma also in dogs can support novel application of somatostatin preparations in the future.^22,30^ However, it is necessary to be aware that radiolabeled somatostatin does not definitely provide the functional status of SSTRs and the response to therapy.^19^ Finally, the recent approval of everolimus use with oral administration by the United States Food and Drug Association for the treatment of adult patients with progressive, nonfunctional, and well-differentiated neuroendocrine tumors of lung or gastrointestinal origin, really calls for novel combined therapeutic protocols to be tested in canine clinical trials in the next future. In this context, the knowledge exchange from human to canine oncology is absolutely necessary to support a One Health approach to the meningioma to further regard this tumor as a substantial animal model for the human counterpart.^5^

## Data Availability

All the reported data are available to the authors.

## References

[1] Belluco S, Marano G, Baiker K, et al. Standardisation of canine meningioma grading: Inter-observer agreement and recommendatons for reproducible histopathologic criteria. Vet Comp Oncol. 2022;20(2):509–520. 35066998 10.1111/vco.12802

[2] Belluco S, Marano G, Luirer T, et al. Standardisation of canine meningioma grading: Validation of new guidelines for reproducible histopathologic criteria. Vet Comp Oncol. 2023;21(4):685–699. 37635372 10.1111/vco.12932

[3] Sahm F, Perry A, Brastianos PK, et al. Meningioma. In: WHO Classificaton of Tumours Editorial Board. Central Nervous System Tumours (5th ed. Louis, D.N.) Lyon (France); 2021:284–297.

[4] Goldbrunner R, Stavrinou P, Jenkinson MD, et al. EANO guideline on the diagnosis and management of meningiomas. Neuro Oncol. 2021;23(11):1821–1834. 34181733 10.1093/neuonc/noab150 PMC8563316

[5] Tomanelli M, Florio T, Vargas GC, Pagano A, Modesto P. Domestic animal models of central nervous system tumors: Focus on meningiomas. Life (Basel). 2023;13(12):2284. 38137885 10.3390/life13122284 PMC10744527

[6] Miller AD, Miller CR, Rossmeisl J. Canine primary intracranial cancer: A clinicopathologic and comparative review of glioma, meningioma, and choroid plexus tumors. Front Oncol. 2019;9(11):1–22. 31788444 10.3389/fonc.2019.01151 PMC6856054

[7] Schwarz P, Meier V, Soukup A, et al. Comparative evaluation of a novel, moderately hypofractionated radiation protocol in 56 dogs with symptomatic intracranial neoplasia. J Vet Intern Med. 2018;32(6):2013–2020. 30308086 10.1111/jvim.15324 PMC6272041

[8] Jung HW, Lee HC, Kim JH, et al. Imatinib mesylate plus hydroxyurea chemotherapy for cerebellar meningioma in a Belgian Malinois dog. J Vet Med Sci. 2014;76(11):1545–1548. 25131949 10.1292/jvms.14-0001 PMC4272992

[9] Andersen BM, Pluhar GE, Seiler CE, et al. Vaccination for invasive canine meningioma induces in situ production of antibodies capable of antibody-dependent cell-mediated cytotoxicity. Cancer Res. 2013;73(10):2987–2997. 23471847 10.1158/0008-5472.CAN-12-3366 PMC3655124

[10] Foiani G, Guelfi G, Chiaradia E, et al. Somatostatin Receptor 2 expression in canine meningioma. J Comp Pathol. 2019;166(1):59–68. 30691608 10.1016/j.jcpa.2018.11.002

[11] Bousquet C, Guillermet J, Vernejoul F, et al. Somatostatin receptors and regulation of cell proliferation. Dig Liver Dis. 2004;36(2):S2–S7. 15077905 10.1016/j.dld.2003.11.007

[12] Lamberts SWJ, Hofland LJ. Anniversary review: Octreotide, 40 years later. Eur J Endocrinol. 2019;181(11):R173–R183. 31398712 10.1530/EJE-19-0074

[13] Mandara MT, Foiani G, Silvestri S, Chiaradia E. Immunoexpression of epithelial membrane antigen in canine meningioma: Novel results for perspective considerations. Vet Comp Oncol. 2021;19(1):115–122. 32875656 10.1111/vco.12648

[14] De Oliveira Silva CB, Ongaratti BR, Trott G, et al. Expression of somatostatin receptors (SSTR1-SSTR5) in meningiomas and its clinicopathological significance. Int J Clin Exper Path. 2015;8(10):13185–13192. 26722517 PMC4680462

[15] Ferjoux G, Bousquet C, Cordelier P, et al. Signal transduction of somatostatin receptors negatively controlling cell proliferation. J Physiol Paris. 2000;94(3–4):205–210. 11087998 10.1016/s0928-4257(00)00206-0

[16] Graillon T, Romano D, Defilles C, et al. Octreotide therapy inmeningiomas: In vitro study, clinical correlation, and literature review. J Neurosurg. 2017;127(3):660–669. 27982767 10.3171/2016.8.JNS16995

[17] Hofland LJ, Lamberts SW. Somatostatin receptors and disease: Role of receptor Subtypes. Baillieres Clin Endocrinol Metab. 1996;10(1):163–176. 8734455 10.1016/s0950-351x(96)80362-4

[18] PubChem Lanreotide. Available at https://pubchem.ncbi.nlm.nih.gov/compound/6918011. Accessed February, 10, 2021.

[19] Miyagishima D, Moliterno J, Claus E, et al. Hormone therapies in meninigioma-where are we? J Neurooncol. 2023;161(2):297–308. 36418843 10.1007/s11060-022-04187-1 PMC10371392

[20] Hicks J, Platt S, Kent M, Haley A. Canine brain tumours: A model for the human disease? Vet Comp Oncol. 2017;15(1):252–272. 25988678 10.1111/vco.12152

[21] Jensen LR, aier AD, Lomstein A, et al. Somatostatin analogues in treatment refractory meningioma: A systematic review with meta-analysis of individual patient data. Neurosurg Rev. 2022;45(5):3067–3081. 35984552 10.1007/s10143-022-01849-6

[22] Chamberlain MC, Glantz MJ, Fadul CE. Recurrent meningioma: Salvage therapy with long-acting somatostatin analogue. Neurology. 2007;69(10):969–973. 17785665 10.1212/01.wnl.0000271382.62776.b7

[23] Johnson DR, Kimmel DW, Burch PA, et al. Phase II study of subcutaneous octreotide in adults with recurrent or progressive meningioma and meningeal hemangiopericytoma. Neuro Oncol. 2011;13(5):530–535. 21558077 10.1093/neuonc/nor044 PMC3093340

[24] Simó M, Argyriou AA, Macià M, et al. Recurrent high-grade meningioma: A phase II trial with somatostatin analogue therapy. Cancer Chemother Pharmacol. 2014;73(5):919–923. 24619496 10.1007/s00280-014-2422-z

[25] Cardona AF, Ruiz-Patiño A, Zataraub-Barròn ZL, et al. Systemic management of malignant meningiomas: A comparative survival and molecular marker analysis between Octreotide in combination with Everolimus and Sunitinib. PLoS One. 2019;14(6):e0217340. doi:10.1371/journal.pone.0217340. 31220093 PMC6586269

[26] Graillon T, Defilles C, Mohamed A, et al. Combined treatment by octreotide and everolimus: Octreotide enhances inhibitory effect of everolimus in aggressive meningiomas. J Neurooncol. 2015;124(1):33–43. 26015296 10.1007/s11060-015-1812-3

[27] Graillon T, Romano D, Defilles C, et al. Pasireotide is more effective than octreotide, alone or combined with everolimus on human meningioma in vitro. Oncotarget. 2017;8(33):55361–55373. 28903425 10.18632/oncotarget.19517 PMC5589664

[28] Graillon T, Sanson M, Campello C, et al. Everolimus and octreotide for patients with recurrent meningioma: Results from the phase II CEVOREM Trial. Clin Cancer Res. 2020;26(3):552–557. 31969329 10.1158/1078-0432.CCR-19-2109

[29] Tollefsen SE, Solheim O, Mjønes P, Torp SH. Meningiomas and somatostatin analogs: A systematic scoping review on current insights and future perspectives. Int J Mol Sci . 2023;24(5):4793. 36902224 10.3390/ijms24054793 PMC10003463

[30] Puchner MJA, Hans VH, Harati A, et al. Bevacizumab-induced regression of anaplastic meningioma. Ann Oncol. 2010;21(12):2445–2446. 21041375 10.1093/annonc/mdq634

